# Target Therapy in Hematological Malignances: New Monoclonal Antibodies

**DOI:** 10.1155/2014/701493

**Published:** 2014-10-29

**Authors:** Monika Podhorecka, Justyna Markowicz, Agnieszka Szymczyk, Johannes Pawlowski

**Affiliations:** ^1^Department of Hematooncology and Bone Marrow Transplantation, Medical University of Lublin, Staszica 11, 20-081 Lublin, Poland; ^2^Students Scientific Association at the Department of Hematooncology and Bone Marrow Transplantation, Medical University of Lublin, Lublin, Poland

## Abstract

Apart from radio- and chemotherapy, monoclonal antibodies (MoAbs) represent a new, more selective tool in the treatment of hematological malignancies. MoAbs bind with the specific antigens of the tumors. This interaction is a basis for targeted therapies which exhibit few side effects and significant antitumor activity. This review provides an overview of the functional characteristics of MoAbs, with some examples of their clinical application. The promising results in the treatment of hematological malignancies have led to the more frequent usage of MoAbs in the therapy. Development of MoAbs is a subject of extensive research. They are a promising method of cancer treatment in the future.

## 1. Introduction

In many cases of hematological malignancies the classical chemotherapy did not show the expected effects. For this reason the scientists have been trying to find a new, more selective therapy, which would target only neoplastic cells. In 1997, for the very first time, a novel treatment was introduced. It was when the FDA approved rituximab, the first anti-CD20 monoclonal antibody (MoAb) [1–3]. It has had such a long lasting impact that you can now come across the term “rituximab era” in the medical literature. Today, targeted therapy, based on selective inhibition of molecules, is becoming increasingly popular [2].

MoAbs, used in hematooncology, are immunoglobulins, which selectively bind molecular targets and suppress carcinogenesis. The first MoAbs were derived from mice and provoked a strong immunological reaction when given to humans. The progress in genetic engineering allowed us to produce chimeric MoAbs, consisting of 60 up to 90% of human antigens. Chimeric MoAbs consist of human constant regions and mouse variable regions, responsible for the recognition of the antigen. Humanized antibodies contain up to 90% of human sequences. They are produced by merging highly variable mice regions, which are responsible for the specificity of the antibody, with human regions [4]. MoAbs used in modern therapy are completely humanized and they contain only human amino acid sequences. MoAbs destroy neoplastic cells in three different ways. They can induce apoptosis and block growth factor receptors or modulate their ligand-receptor interaction. In order to reach high therapeutic goals, MoAbs are conjugated with radioisotopes, toxins, cytostatics, or cytokines [4].

This review will focus on three major aspects of monoclonal antibody therapy: (1) brief description of monoclonal antibodies used in hematooncology, (2) new therapeutic approaches to currently approved agents, and (3) preclinical and clinical experience with new agents in the last few years.

## 2. Monoclonal Antibodies Used in Hematology

Several studies in the fields of molecular biology, biochemistry, and genetics proved that there are a number of molecular signaling pathways and molecules, whose selective inhibition or modification leads to the elimination of the neoplastic cells. Currently, several MoAbs are FDA approved for the therapy of hematological diseases (Table 1). Their short description is presented below.

### 2.1. Rituximab

Rituximab has been approved for the treatment of non-Hodgkin's lymphoma (NHL) by the FDA in 1997. From that moment on a new era, called “rituximab era,” began. Since that time, many other antibodies, whose target is the CD20 antigen, have been introduced. Currently, rituximab is widely used to treat a number of diseases caused by an abnormal production of B cells. It is approved by the FDA for the treatment of B-cell non-Hodgkin's lymphomas, including chronic lymphocytic leukemia (CLL) and rheumatoid arthritis [5]. It is also used in the treatment of autoimmune hemolytic anemia, graft-versus-host disease, chronic immune-mediated thrombocytopenia, posttransplant lymphoproliferative disorder, pemphigus vulgaris, systemic lupus erythematosus, multiple sclerosis, and Evans Syndrome [5, 6].

### 2.2. Ibritumomab

Ibritumomab is a murine IgG-1 kappa anti-CD20 antibody that is conjugated to yttrium-90 (pure beta emitter) or indium-111 (gamma emitter) in the presence of chelate tiuxetan (Mx-DTPA) [7, 8]. This monoclonal antibody binds to the CD20 antigen found on the surface of malignant and normal B cells. Radiation from the attached isotope kills malignant cells. Antibodies can also cause cell's death by antibody-dependent cell-mediated cytotoxicity (ADCC), complement-dependent cytotoxicity (CDC), and apoptosis.

The main indication for ibritumomab therapy is relapsed or refractory, low grade or follicular, B-cell NHL [9]. In 2009 ibritumomab was also approved for consolidation therapy in patients with follicular lymphoma who achieved response to first-line chemotherapy. Contraindications for the use of ibritumomab include absolute neutrophil count lower than 1500 cells/mm³ or platelet count lower than 100,000/mm^3^ or effective beam radiation therapy of >25 percent of active marrow. It must not be used in patients who had prior autologous stem cell transplantation, during pregnancy or ongoing breastfeeding, and in patients with allergy or hypersensitivity to the murine antibodies or components of the therapy [8].

### 2.3. Ofatumumab

Ofatumumab is an IgG1 (kappa) fully human monoclonal antibody whose epitope on CD20 is distinct from rituximab's target and activates CDC more effectively. It induces cell lysis of both high and low CD20-expressing cells and of rituximab-resistant cells [6, 10].

An initial phase I/II study of 33 patients with CLL who received 4, once weekly, infusions of ofatumumab showed a 50% overall response rate [10]. Promising results encouraged researchers to continue the study in a larger group of patients. In 2008, during the conference of the American Society of Hematology, Wierda et al. [11] presented the results of a phase II clinical trial. The study group consisted of patients with relapsed CLL refractory to fludarabine and alemtuzumab (response rate 58%) or with bulky lymphadenopathy refractory to fludarabine (response rate 47%). Clinical improvement was achieved for several symptoms, such as lymphadenopathy, splenomegaly, and hematological parameters. Based on these results, the FDA and EMEA registered ofatumumab for the treatment of patients with CLL in 2010 [6, 11]. The recent study comprised 77 previously untreated patients, who were divided into two groups and received either 2000 mg or 1000 mg of ofatumumab as an induction therapy, once a week [12]. Maintenance therapy was continued at the same dosage for a consecutive period from two months to two years. The overall response rate for the high doses group and the low doses group was 55% and 36%, respectively. Recently, there have been some reports about two studies in which ofatumumab was combined with lenalidomide, a drug that augments and sensitizes CLL cells to monoclonal antibody-induced cell death. In one of these studies 36 patients with CLL received ofatumumab once a week for 4 weeks, once a month during months 2–6, and every other month during months 7–24. Lenalidomide was administered on day 9 of every month of the treatment. The objective overall response rate in 34 evaluable patients was 68% and median duration of response was 22 months. The second phase of the study evaluated the efficacy of the same treatment regimen in high-risk patients. The therapy was well tolerated and the most common side effect was neutropenia [13, 14].

Ofatumumab was also studied in patients with follicular lymphoma refractory to rituximab. Overall response rate was 10% for patients receiving the dose of  1000 mg of ofatumumab weekly and 13% for the group receiving the dose of 500 mg weekly. The most common side effects were infections, rash, urticaria, fatigue, and pruritus. Severe adverse events such as neutropenia, anemia, or thrombocytopenia were less common [15, 16].

### 2.4. Tositumomab

This radioimmunopharmaceutical was introduced by the FDA in 2003. It is a murine IgG2a anti-CD20 antibody, which has been linked to radioactive iodine, and emits gamma radiation. It is characterized by a relatively long half-life of 193 hours. The maximum range in the healthy tissue for tositumomab is only 2.3 mm and for this reason its use as a myelosuppressive agent is limited [8]. The underlying cause is the accumulation of antibodies which emit anti-gamma radiation in the tissue. Several studies have reported that in the group of patients previously treated with radioimmunoassay myelodysplastic syndromes are more frequent (2.5–3%) than in other groups, but it is difficult to clearly assess whether it is the effect of radioactive substances or chemotherapy. Tositumomab is concentrated in the thyroid gland and may cause its hypofunction. Administration of potassium iodide during the treatment can prevent this side effect. Compared with ibritumomab tiuxetan, the radiation dose accumulated in the liver is lower but the dose accumulated in kidneys is higher. The main indication for the treatment with tositumomab is refractory, CD20-positive, low-grade, follicular, or transformed NHL in patients refractory to rituximab treatment. In the group of previously untreated patients with low-grade lymphoma the response rate was 100% while the complete response rate was 56% [17–19].

### 2.5. Gemtuzumab

Gemtuzumab ozogamicin is an engineered humanized monoclonal IgG4 antibody directed against the CD33 antigen present on leukemic cells in 80% of patients with AML. In the group of patients responding to gemtuzumab >90% had very high CD33 antigen expression. This monoclonal antibody is linked to calicheamicin, which is a cytotoxic antibiotic attached to the antibody through a covalent linkage. Thanks to this condensation calicheamicin is stable in physiologic buffers and is efficiently released inside lysosomes [20, 21]. 40 patients with morphological evidence of leukemia in the bone marrow were treated with gemtuzumab in the phase I trial. After 1 to 3 doses 20% of these patients had <5% leukemic blast cells in the bone marrow [22].

Further multicenter trials, which comprised 142 patients with AML in first relapse, have shown that gemtuzumab was effective in thirty percent of the patients [23]. Based on these pivotal studies, in 2000, gemtuzumab was registered by the FDA for the treatment of patients with CD33-positive AML in first relapse who were 60 years of age or older and who were not considered candidates for cytotoxic chemotherapy. Gemtuzumab can be also used in patients with relapsed acute promyelocytic leukemia. In this type of AML prolonged molecular remissions were obtained when gemtuzumab was used in monotherapy and when it was administered in combination with other drugs [24].

However, the next study, SWOG S0106, did not confirm the clinical benefits of using gemtuzumab in combination with daunorubicin and cytarabine. The most common adverse events in this trial, that is, infection, hemorrhage, and ARDS, were reported more frequently when gemtuzumab was used in combination with other drugs than in the monotherapy. A second phase III trial, evaluating the addition of gemtuzumab to induction and/or consolidation therapy in the first-line treatment, did not confirm the clinical benefits of gemtuzumab either. Based on these results, in October 2010, gemtuzumab was withdrawn from the US and European markets, but it is still available in Japan [23, 24].

The most common adverse reaction associated with the use of gemtuzumab was hepatic injury (the increased transaminases and bilirubin level). An increased incidence of venoocclusive disease (VOD) in the group of these patients was also reported. It is estimated that this type of complication after allo-HSCT was observed in 0–70% of cases, and the risk of these adverse events increases in the group of patients with liver damage, in which myeloablative chemotherapy or busulfan therapy was used [25]. Thus, the use of this MoAb in clinical practice needs further investigation.

### 2.6. Brentuximab

Brentuximab is a chimeric IgG1 antibody that targets the cell membrane protein CD30, expressed on Hodgkin lymphoma cells and anaplastic large-cell lymphoma cells. It is linked through a peptide linker to the potent inhibitor of microtubule polymerization, monomethylauristatin E (MMAE) [26, 27].

In phase I studies brentuximab was evaluated in a group of 45 patients with relapsed or refractory CD30-positive malignancies. The drug was administered in the doses ranging from 0.1 to 3.6 mg/kg given every 3 weeks by intravenous infusion. The adverse events depended on the treatment dose and the most common included fatigue, fever, diarrhea, nausea, neutropenia, and peripheral neuropathy, headache, vomiting, back pain, anemia, and alopecia. Tumor regression was reported for 83% of patients [28].

In phase II studies of brentuximab the group of 102 patients with relapsed or refractory Hodgkin lymphoma was analyzed. Patients were administered the dose of 1.8 mg/kg of this monoclonal antibody every three weeks. Seventy-five percent of these patients achieved an objective response. The results were reported at the 2011 Annual Meeting of the American Society of Clinical Oncology [27].

In April 2010, a double-blind, placebo-controlled, phase III study was initiated on patients with posttransplant Hodgkin lymphoma. This study is expected to be completed soon. On 14 July 2011, Food and Drug Administration approved brentuximab for the treatment of Hodgkin lymphoma relapsed after autologous stem cell transplantation and for the management of relapsed anaplastic large-cell lymphoma. The results of the phase III trial will form the basis for full FDA approval [26].

### 2.7. Alemtuzumab

Alemtuzumab is a humanized anti-CD52 monoclonal antibody, which is derived from the original rat antibody, Camppath-1 G [29]. It is highly effective in the elimination of cells of the B- and T-lymphocyte lineages, both normal and malignant. Alemtuzumab might be given intravenously as well as subcutaneously. The injection often induces an immune response with clinical signs such as chills, fever, or erythema. These reactions are associated especially with the release of the cytokines: TNF-*α*, INF-*γ*, and IL-6 [30].

Alemtuzumab is indicated in the therapy of CLL, T-cell prolymphocytic leukemia, cutaneous T-cell lymphoma, and peripheral T-cell lymphoma [31]. Alemtuzumab is associated with viral (e.g., CMV or VZV) and fungal opportunistic infections. For this reason it is advisable to carefully assess each patient before the therapy is started [30]. Alemtuzumab reduces the number of B- and T-lymphocytes, thus causing immunosuppression. Lymphopenia is sometimes profound and long-lasting; therefore, it is recommended to use anti-infective prophylaxis with cotrimoxazole and acyclovir for 6 months after completing alemtuzumab therapy [32].

The first clinical trials of alemtuzumab in patients with chronic lymphoid leukemia were performed in 1997. Further studies were carried out by Lundin et al. and they included a much bigger group of patients [30]. In this group 33% of patients responded to alemtuzumab (lymphocyte count decreased rapidly), but significant decrease in lymphadenopathy was observed in only 7% of them [30].

A phase II clinical trial examined the efficiency of alemtuzumab given subcutaneously in relapse patients with progressive form of CLL. The response rate was 87%, including 19% of complete remissions. The FDA approved alemtuzumab for the treatment of CLL in patients with resistance to fludarabine and alkylating agent therapy [30].

## 3. Monoclonal Antibodies Used in Clinical Trials or Preclinical Development

Currently, many MoAbs are being studied in clinical trials and their use in the treatment of hematological diseases still develops. The aim of this review article is to describe only the most important of them.  Table 2 presents their short description.

### 3.1. Apolizumab

Apolizumab (Hu1D10), a humanized monoclonal anti-human leukocyte antigen- (HLA-) DR beta-chain antibody, is currently studied in clinical trials for hematologic malignancies [31]. The HLA-DR antigen is selectively expressed on immune cells, such as lymphocytes, monocytes, and dendritic cells [33]. Hu1D10 induces complement-mediated cytotoxicity, antibody-dependent cell-mediated cytotoxicity [34], and caspase-independent apoptosis following secondary cross-linking in CLL cells [35]. Preclinical and early clinical studies suggest that apolizumab has some activity in CLL and NHL patients with acceptable toxicity profiles [36, 37]. It can be safely administered as a slow intravenous infusion but it causes severe toxicity in animal models when given as a bolus [34]. Granulocyte colony-stimulating factor (G-CSF) treatment significantly enhanced lymphoma cell killing by HLA class II antibodies, including apolizumab [38]. Another study assessed the effects of apolizumab in combination with rituximab in patients with relapsed/refractory NHL. Preliminary results of this phase I clinical trial suggest that this combination may be more effective than rituximab alone [39].

### 3.2. Milatuzumab

Milatuzumab is a new humanized antibody which targets tumors with CD74 antigen, expressed on monocytes, macrophages, and B cells but not on T cells [40]. It has recently been shown that binding of milatuzumab to the CD74 antigen on human peripheral B-cell subsets causes a number of effects, including inhibition of proliferation, enhanced spontaneous migration, alterations of adhesion molecule expression, and CXCL12-dependent chemotaxis of B cells [41, 42]. These biological functions combined with the expression of CD74 on malignant B cells and limited expression on normal tissues implicate CD74 as a potential therapeutic target [40]. Milatuzumab is the first anti-CD74 antibody that has entered into clinical tests and is currently being studied for the treatment of NHL, CLL, and multiple myeloma (MM) [43–45]. Broad expression and fast internalization make CD74 an ideal target for the delivery of chemotherapeutic agent against CD74-expressed tumors [46, 47]. Milatuzumab, as a doxorubicin conjugate, is currently in a phase I/II clinical trial for the treatment of patients with relapsed MM and in combination with rituximab represents a potential therapeutic strategy for the treatment of mantle cell lymphoma (MCL) patients [48, 49]. Another preclinical study shows that milatuzumab can enhance the therapeutic efficacy of bortezomib, doxorubicin, and dexamethasone in MM cell lines [50]. There is possible synergy in action between milatuzumab and another MoAb—veltuzumab [51]. The ongoing trials testing different doses and treatment schedules of milatuzumab in CLL, NHL, and MM showed no severe adverse effects in humans [52].

### 3.3. Monoclonal Antibodies against CD22 Antigen: Inotuzumab and Epratuzumab

The antigen CD22 is a transmembrane glycoprotein expressed on mature B cells and on up to 90% of B malignant cells [53]. Its function is unclear; however, recent studies suggest that it regulates B-cell functions, has an impact on their survival, and serves as an adhesion molecule [54]. Therefore, CD22 is a candidate target for therapeutic antibodies. In this review we will focus on two novel anti-CD22 MoAbs, inotuzumab and epratuzumab, which are currently under investigation in B-cell malignancies.

Inotuzumab ozogamicin (CMC-544) is an antibody-drug conjugate composed of a humanized anti-CD22 antibody linked to calicheamicin, a potent cytotoxic agent [22]. This linkage enables direct intracellular delivery of the chemotherapeutic agent, which reduces toxicity and normal tissue exposure [55]. CD22 binding causes rapid drug internalization and results in DNA damage and cellular apoptosis [56].

Clinical trials revealed inotuzumab ozogamicin activity in the treatment of B-cell NHL, including follicular lymphoma and diffuse large B-cell lymphoma (DLBCL), in patients who failed prior therapies [57]. It was effective in monotherapy, while combination with rituximab increased its antitumor activity [58]. Moreover, some studies suggest that inotuzumab ozogamicin successfully induces remission in patients with relapsed acute lymphoblastic leukemia allowing more patients to receive stem cell transplant (SCT) [56, 59–61]. The most frequently described adverse events in inotuzumab ozogamicin treatment were thrombocytopenia, neutropenia, and hepatotoxicity [22, 62]. Combination with rituximab did not significantly change drug safety profile [63].

Epratuzumab is another humanized anti-CD22 MoAb. Similarly to inotuzumab it demonstrated the activity in B-cell NHL patients who have relapsed or are refractive to conventional therapy, including rituximab [64]. Epratuzumab has been evaluated as a single agent or in combination with rituximab or standard chemotherapy [65]. Several studies revealed that combination of epratuzumab and rituximab in NHL patients enhances clinical efficacy and was better tolerated than rituximab alone [66, 67]. Therefore, bispecific MoAbs consisting of veltuzumab (humanized anti-CD20) and epratuzumab (humanized anti-CD22) were constructed and evaluated [68]. A promising pilot study described chemotherapy based on epratuzumab with rituximab, cyclophosphamide, doxorubicin, vincristine, and prednisone (R-CHOP) in untreated DLBCL [69]. Epratuzumab has antilymphoma activity in both, unlabeled and radiolabeled, forms. Recently the phase I/II trials with fractionated radiolabeled anti-CD22 MoAb have been published and they showed high rates of durable CR in recurrent and heavily pretreated NHL patients [70]. Moreover, the drug is currently under investigation as a novel targeted therapy in acute lymphoblastic leukemia (ALL) and autoimmune diseases such as systemic lupus erythematosus and primary Sjögren's syndrome [71–73]. Some data demonstrate that this agent has good safety profile without dose-dependent toxicity [74]. No drug interaction has yet been reported with epratuzumab [75]. Infusion-connected toxicity appears to be less intense than for rituximab [76]. Because epratuzumab has a different effect on cell growth in comparison to rituximab it may become an important component of the future therapies for NHL.

### 3.4. Obinutuzumab

Obinutuzumab (GA101) is a glycoengineered, IgG1, anti-CD20 monoclonal antibody that is classified as type II MoAb [77, 78]. It induces cell death through caspase-independent way and has shown lower levels of CDC [77]. In phase I/II trials obinutuzumab was evaluated as a monotherapy in 12 patients with malignant diseases with CD20 expression. The complete response was obtained in 25% of patients and partial response in 33% of them [77, 79].

Obinutuzumab and chlorambucil combination is now evaluated in a phase III study of patients with previously untreated CLL. It is estimated that the study will be completed in February 2022 [77, 79]. Another study is assessing obinutuzumab and bendamustine combination therapy in patients with rituximab-refractory indolent NHL. Perhaps this study will be completed in January 2015 [77].

Obinutuzumab or rituximab and chlorambucil combination was evaluated in a phase III study of patients with previously untreated CLL. This study compared the effectiveness of chlorambucil monotherapy with combination therapy of rituximab with chlorambucil and obinutuzumab with chlorambucil. It has been shown that patients who used combination therapy achieved a longer disease-free period (26.7 versus 11.1 months) and a higher percentage of complete response to treatment. The application of obinutuzumab and chlorambucil compared with rituximab and chlorambucil resulted in a prolongation of progression-free survival and the number of overall responses (20.7% versus 7%) and molecular responses. The infusion-related reactions and neutropenia during obinutuzumab therapy were observed more frequently. Nevertheless the increased risk of infection was not shown [80].

### 3.5. Veltuzumab

Veltuzumab (HA20) is a humanized type I, IgG1, anti-CD20 MoAb constructed on the framework regions of epratuzumab. It has a stronger impact on CDC than rituximab but has shorter infusion time [81]. In the clinical trial phase I/II study veltuzumab was administered to a group of 82 patients with relapsed/refractory B-cell NHL. MoAb was administered intravenously at the doses of 80–750 mg/m^2^, once weekly for four doses. As many as 27% of patients obtained complete response, and the median time of response was 19.7 months [6, 82]. Other studies have reported on the effectiveness of the subcutaneous injections of low doses (80–320 mg): this route of administration seems to be as good as the intravenous one [82]. Further research is carried out on the use of this antibody in the treatment.

### 3.6. Ocrelizumab

Ocrelizumab is a type I, second generation, chimeric, IgG1, anti-CD20 monoclonal antibody, which mediates cell lysis by apoptosis, antibody-dependent cellular cytotoxicity, and complement-dependent cytotoxicity [6, 83, 84]. It is characterized by increased binding affinity for the low-affinity variants of the Fc*γ*RIIIa receptor; therefore it may have greater clinical efficacy in the 80%–85% of patients who are carriers of the low-affinity variants [6]. In trial phase I/II study it was tested in forty-seven patients with relapsed follicular lymphoma after prior rituximab therapy. The single-agent therapy with ocrelizumab resulted in 38% overall response rate [83, 85]. The incidence of infusion-related adverse events was very low. The grade 3/4 toxicity was observed only in 9% of patients, but infusion-related reactions were much more frequent and were observed in 74% of the analyzed patients [6, 85].

### 3.7. Lumiliximab

Lumiliximab is a chimeric monoclonal antibody directed against CD23 antigen, which is highly expressed on CLL cells but only minimally on other cells [86]. Lumiliximab induces apoptosis by activating caspases and downregulating antiapoptotic proteins [87]. It has been investigated as a monotherapy or in combination with rituximab or other chemotherapeutic agents in relapsed or refractory CL.

Clinical data from a phase I dose-escalation trial with lumiliximab monotherapy in pretreated CLL patients demonstrated acceptable toxicity profile when the drug was given 3 times per week for 4 weeks at doses of up to 500 mg/m^2^ [88]. A phase I/II multicenter study revealed synergy of lumiliximab with fludarabine, cyclophosphamide, and rituximab (L + FCR) therapy in the above-mentioned group. The addition of lumiliximab enhanced therapeutic effect of FCR treatment and resulted in high overall response rate and complete remission rate (65%, 52%, resp.). Combination of these drugs showed no additional toxicity [89]. CD23 glycoprotein, as a receptor for IgE, is involved in the regulation of IgE-mediated inflammatory process. Preliminary studies suggested that lumiliximab might provide clinical benefit in patients with persistent allergic asthma and other allergic diseases [90, 91]. The phase III study comparing FCR and FCR plus lumiliximab in relapsed CLL did not confirm the beneficial effects of this combination [92].

### 3.8. Galiximab

Galiximab, anti-CD80 chimeric MoAb, represents a new therapeutic approach to NHL patients. CD80 is a costimulatory molecule involved in T-cell activation [93]. Surface CD80 is transiently expressed on antigen-presenting cells and normal B cells and constitutively expressed on a variety of B-cell lymphomas [94]. After CD80 activation cell death via antibody-dependent cell-mediated cytotoxicity (ADCC) was induced [95]. Moreover, galiximab inhibits tumor cell proliferation through influence on intracellular pathways such as NF-*κ*B pathway [96]. Preclinical studies revealed its significant antitumor activity as a single agent or in combination with rituximab against various B-cell lymphoma cell lines including follicular lymphoma [97]. Despite being well tolerated, galiximab had minimal activity in pretreated patients with relapsed Hodgkin lymphoma (HL) [98]. Phase I/II monotherapy trial of galiximab in relapsed/refractory follicular lymphoma revealed its efficacy and safety profile. The overall response rate was 11% and the most commonly observed adverse events were fatigue, nausea, and headache. Cytopenia occurred rarely [99]. Galiximab showed some promising results when used in combination with rituximab. Phase II trial of galiximab plus rituximab (CALGB trial) in untreated follicular lymphoma showed that the ORR was 72.1% including a 47.6% CR rate [100]. Due to its immunomodulatory properties, galiximab was evaluated as a potential novel therapy for psoriasis [101].

### 3.9. Lintuzumab

Lintuzumab is a humanized monoclonal antibody which targets CD33 molecule. It has been under investigation as a treatment option for myeloid malignancies in patients who do not qualify for intensive chemotherapy or bone marrow transplantation. Lintuzumab, as a single agent, presents minimal antileukemic activity in patients with relapsed/refractory acute myelogenous leukemia (AML) and more significant activity against minimal residual disease in acute promyelocytic leukemia (APL) [102]. The efficacy of lintuzumab was evaluated in combination with induction chemotherapy in relapsed or primary refractory AML. Although the addition of lintuzumab was safe, it did not significantly improve survival rate [103]. A phase II randomized clinical study of lintuzumab in combination with a low dose of cytarabine in elderly adults with untreated AML presented similar results [104]. Recently, it has been reported that 5-azacytidine enhances its mediated effector functions in vitro and induces antitumor effects in vivo [104]. These findings required further clinical investigation. To increase drug potency lintuzumab was conjugated to radionuclides as, for example, actinium (^225^Ac), bismuth (^213^Bi), or iodine (I-131), with promising results in AML patients [105–107]. Moreover, lintuzumab conjugated with immunotoxin recombinant gelonin (HUM-195/rGEL) has been under investigation in patients with relapsed/refractory myeloid leukemias [108].

### 3.10. Monoclonal Antibodies against CD40 Antigen: Dacetuzumab and Lucatumumab

Dacetuzumab is a new humanized anti-CD40 monoclonal antibody in early phase of clinical trials. The CD40 protein is a member of the TNF-receptor superfamily highly expressed on normal B cells [109]. The CD40 signaling may play a role in the development of B-cell hematological malignancies; therefore anti-CD40 MoAb is an attractive option for targeted therapy [110]. Dacetuzumab has been reported to induce antitumor activity against several B-cell lymphomas and MM cell lines in vitro [111]. Dacetuzumab as a monotherapy in NHL patients was well tolerated (doses up to 8 mg/kg/wk) without dose-limiting toxicity [112]. The observed safety profile suggested that combination of dacetuzumab with other chemotherapeutic agents may improve its clinical effectiveness. Preclinical studies revealed synergistic activity between dacetuzumab, gemcitabine, and rituximab in NHL [113]. The combination of dacetuzumab with lenalidomide in MM cells in vitro had synergic effect as well [114]. Moreover, dacetuzumab demonstrated activity that is enhanced by lenalidomide in CLL [115]. Many of these combinations are now being tested clinically. A phase I safety/efficacy study of dacetuzumab in combination with rituximab and gemcitabine was conducted in relapsed/refractory diffuse large B-cell lymphoma (DLBCL) [113]. Two trials are ongoing in patients with relapsed MM to evaluate dacetuzumab in combination with lenalidomide or bortezomib [116, 117].

Lucatumumab is another humanized monoclonal antibody targeting CD40 protein. The drug has two distinct mechanisms of anticancer activity. It blocks the interaction between CD40 and its ligand on malignant cells and it mediates antibody-dependent cell toxicity (ADCC) [118]. In preclinical testing lucatumumab showed antilymphoma activity in vivo and decrease in proliferation of B cells in vitro [119]. It has been already clinically evaluated for treatment of various hematological malignancies. In patients with relapsed CLL tolerability of lucatumumab was acceptable but it had only minimal activity as a single agent [120]. Similarly, in patients with relapsed or refractory MM lucatumumab displayed modest clinical activity [121]. Those findings resulted in subsequent studies of lucatumumab as a combination therapy. Lucatumumab plus bendamustine has been in phase I/II trial for follicular lymphoma patients [122]. Moreover, clinical efficiency of the drug is currently under investigation in patients with relapsed/refractory HL or NHL [123]. In June 2014 the report concerning its effects in the treatment of NHL and HL appeared, which were a continuation of the phase Ia/II study [124]. Patients with diagnosed lymphoma were administered lucatumumab intravenously once a week for 4 weeks of an 8-week cycle. Response was assessed separately for the different subtypes of lymphoma. The overall response rate assessed by computed tomography among patients with marginal zone lymphoma of mucosa-associated lymphatic tissue (MZL/MALT) was 42.9% but, with follicular lymphoma (FL), was 33.3%. Future efforts with lucatumumab should focus on combination-based therapy. Clinical trials on the use of lucatumumab in the treatment of CLL or MM are not conducted further [124].

### 3.11. Blinatumomab

Blinatumomab is the first of the bispecific T-cell engagers (BiTE) antibodies—a new group of agents combining targeted therapy with immunologic activation of T cells, which exert cytotoxic activity on the target cells [125]. Blinatumomab has dual specificity for CD19 and CD3. It links T cells to target cells expressing CD19, a protein found on the majority of B-cell malignancies, causing redirected lysis of tumor cell [126, 127]. The effects of blinatumomab have been evaluated in preclinical studies. Blinatumomab compared with rituximab showed higher degree of lysis of human lymphoma lines and the combination of both drugs enhanced the antitumour activity of blinatumomab [128]. Blinatumomab has received orphan drug designation from the Food and Drug Administration (FDA) and is currently being investigated for the treatment of relapsed/refractory B-cell ALL and relapsed NHLs [129]. Phase II study evaluating the efficacy and safety of blinatumomab in adult patients with ALL showed high rate of complete responses which was 72%. The most common adverse events were pyrexia, headache, tremor, and fatigue. These adverse events occurred most often at the onset of the treatment, during the first course [130]. Blinatumomab was described as effective and well tolerated in patients with minimal residual disease positive- (MRD-) ALL and induced an 80% MRD eradication rate [131]. A long-term follow-up revealed that the hematologic relapse-free survival was 61% of the entire evaluable study cohort [132]. Already published data are based on clinical trials with ALL adults. Recently, multicenter study with blinatumomab in children has been initiated to evaluate the safety profile in all pediatric age subsets [129].

### 3.12. Bevacizumab

Bevacizumab is a humanized monoclonal antibody against vascular endothelial growth factor (VEGF) with a potential to reduce tumor angiogenesis and inhibit tumor growth [133]. So far, the drug has been FDA-approved as a single agent and with combination with chemotherapy for the use in patients with several types of solid tumors, such as colorectal cancer, breast cancer, non-small cell lung cancer, renal cell cancer, and glioblastoma [134]. However, it was shown that cardiovascular toxicity increases especially during combination therapy. Balancing the risks and benefits of bevacizumab requires understanding the spectrum of bevacizumab toxicities and predisposing factors [135]. Bevacizumab has been recently introduced to hematology and its therapeutic effect has been demonstrated in AML, NHL, and HL [136–138]. There is evidence that angiogenesis may be a target in certain lymphoproliferative disorders. Increased VEGF expression has been found in many types of tumors, including NHL [139, 140]. Additionally, elevated VEGF level is associated with adverse prognosis in leukemia and lymphoma patients [141, 142]. Efficacy and safety of bevacizumab in distinct hematological malignancies are still uncertain and require further investigation. Bevacizumab as a single agent has been reported to have modest clinical activity in patients with relapsed aggressive NHL [137]. Clinical trial of standard R-CHOP therapy with bevacizumab in patients with untreated DLBCL showed that the combination was safe and well tolerated [143]. However, for patients with newly diagnosed DLBCL the combination of drugs did not significantly prolong progression-free survival and resulted in increased serious toxicities [144]. Bevacizumab in combination with standard (3 + 7) chemotherapy has been also evaluated in elderly patients with AML, but it did not improve the therapeutic outcomes [135]. On the other hand, cytotoxic chemotherapy with cytarabine and mitoxantrone followed by bevacizumab revealed a favorable CR rate and its duration in adults with AML resistant to traditional treatment [136]. Moreover, bevacizumab is currently under investigation as a treatment option for newly diagnosed AML in combination with idarubicin and cytarabine [145]. In vivo and in vitro studies showed that bevacizumab has an ability to enhance the chemotherapeutic effect on T-leukemia/lymphoma cells [146, 147]. In the case of HL, bevacizumab caused biological effects, which indicate it is clinically active [138].

## 4. Conclusions

For many years, MoAbs have been a well-known and constantly improved option for the targeted therapy of many hematological diseases. In recent years, technological progress allowed construction of MoAbs other than their classical forms: for example, conjugated with radionucleotides, immunotoxins, or bispecific antibodies. The new studies provide us with more and more encouraging results that open up new prospects for the usage of monoclonal antibodies in clinical practice.

## Figures and Tables

**Table 1:** Monoclonal antibodies clinically used currently.

Agent	Name	Target	Type	FDA approval	Indication	Selected side effects	Clinical application
Rituximab	Rituxan	CD20	Chimeric	1997	B-cell NHL, CLL	Infusion related, fever, lymphopenia, neutropenia, susceptibility to infections, and progressive multifocal leukoencephalopathy	Agents currently clinically used

Ibritumomab	Zevalin	CD20	Mouse/radiolabeled	2002	B-cell NHL	Cytopenias, fatigue, nasopharyngitis, nausea, abdominal pain, asthenia, cough, diarrhea, and pyrexia	Agents currently clinically used

Tositumomab	Bexxar	CD20	Mouse/radiolabeled	2003	NHL	Neutropenia, thrombocytopenia, anemia, infections, infusion reactions, asthenia, fever, and nausea	Agents currently clinically used

Ofatumumab	Arzerra	CD20	Human	2009	CLL	Neutropenia, pneumonia, pyrexia, cough, diarrhea, anemia, fatigue, dyspnea, rash, nausea, bronchitis, and upper respiratory tract infections	Agents currently clinically used

Gemtuzumab	Mylotarg	CD33	Humanized/drug attached	2000	Relapsed AML	Infusion related, skin, and hepatotoxicity including the syndrome of venoocclusive disease	Withdrawn

Brentuximab	Adcetris	CD30	Chimeric/chemolabeled	2011	HL, sALCL	Neutropenia, peripheral sensory neuropathy, fatigue, nausea, anemia, upper respiratory infection, diarrhea, fever, cough, vomiting, and thrombocytopenia	Agents currently clinically used

Alemtuzumab	Campath	CD52	Humanized	2001	B-CLL	Cytopenias, infusion reactions, cytomegalovirus (CMV) and other infections, nausea, emesis, diarrhea, and insomnia	Agents currently clinically used

Eculizumab	Soliris	C5	Humanized	2007	PNH, aHUS	Hypertension, upper respiratory tract infection, diarrhea, headache, anemia, vomiting, nausea, urinary tract infection, and leucopenia	Agents currently clinically used

NHL: non-Hodgkin's lymphoma; CLL: chronic lymphocytic leukemia; AML: acute myelocytic leukemia; HL: Hodgkin's lymphoma; sALCL: systemic anaplastic large cell lymphoma; PNH: paroxysmal nocturnal hemoglobinuria; aHUS: atypical haemolytic uremic syndrome.

**Table 2:** New monoclonal antibodies under investigation in clinical trials.

Agent	Target	Type	Suggested indication	Clinical trials
Milatuzumab	CD74	Humanized	CLL, MM, NHL	(i) Phase I/II Study of Veltuzumab Combined with Milatuzumab in Relapsed and Refractory NHL (ii) Phase I/II Study of hLL1-DOX (Milatuzumab-Doxorubicin Antibody-Drug Conjugate) in Patients with Relapsed NHL and CLL

Apolizumab	HLA-DR beta	Humanized	CLL, NHL	No currently active clinical trials found

Epratuzumab	CD22	Humanized	NHL, ALL, WM	(i) Phase I/II Study of Veltuzumab Combined with 90Y-Epratuzumab Tetraxetan in Patients with Relapsed/Refractory, Aggressive NHL (ii) International Study for Treatment of Standard Risk Childhood Relapsed ALL 2010: A Randomized Phase III Study Conducted by the Resistant Disease Committee of the International BFM Study Group (iii) Phase I/II Study Combining Humanised Anti-CD20 (Veltuzumab), Anti-CD22 (Epratuzumab) and Both Monoclonal Antibodies with Intensive Chemotherapy in Adults with Recurrent or Refractory B-Precursor ALL (iv) Evaluation of the Efficacy and Tolerance of Fractionated Radio-immunotherapy with 90Y-Epratuzumab (90Y-hLL2) for Relapsed or Refractory CD22+ B-ALL

Veltuzumab	CD20	Humanized	NHL, ITP, CLL, ALL	(i) Phase I/II Study of Veltuzumab Combined with 90Y-Epratuzumab Tetraxetan in Patients with Relapsed/Refractory, Aggressive NHL (ii) A Phase I/II Study of Veltuzumab (IMMU-106, hA20), a Humanized Anti-CD20 Monoclonal Antibody, Combined with Milatuzumab (IMMU-115, hLL1), a Humanized Anti-CD74 Monoclonal Antibody in Relapsed and Refractory B-Cell NHL (iii) A Phase I/II Study of Immunotherapy with Humanized Anti-CD20 Antibody, IMMU-106 (hA20), in Adult Patients with Chronic ITP (iv) Phase I/II Study Combining Humanised Anti-CD20 (Veltuzumab), Anti-CD22 (Epratuzumab) and Both Monoclonal Antibodies with Intensive Chemotherapy in Adults with Recurrent or Refractory B-Precursor ALL

Lumiliximab	CD23	Chimeric	CLL	No currently active clinical trials found

Galiximab	CD80	Chimeric	NHL	(i) A Phase II Study of Galiximab (Anti-CD80) for Patients with Relapsed/Refractory NHL (ii) A Phase II Trial of Extended Induction Galiximab (Anti-CD80 Monoclonal Antibody) (IND #XXXXX) Plus Rituximab in Previously Untreated Follicular NHL

Bevacizumab	VEGF	Humanized	MM, HL	(i) Phase II Trial of Bevacizumab Combined with Lenalidomide and Dexamethasone (BEV/REV/DEX) in Relapsed or Refractory MM (ii) Avastin (Bevacizumab) in Combination with ABVD for the Treatment of Newly Diagnosed Advanced Stage Classical HL

Obinutuzumab	CD20	Humanized	CLL, NHL	(i) Open-Label, Multicenter, Three-Arm Randomized Study to Investigate the Safety and Efficacy on Progression-Free Survival of RO5072759 + Chlorambucil (GClb) Compared to Rituximab + Chlorambucil (RClb) or Chlorambucil (Clb) Alone in Previously Untreated CLL Patients with Comorbidities (ii) Open-Label, Multicenter, Randomized Study to Evaluate the Efficacy on Tumor Response of GA101 (RO5072759) Monotherapy versus Rituximab Monotherapy in Patients with Relapsed CD20+ Indolent NHL (iii) An Open-Label, Multicenter, Three-Arm Randomized Study to Investigate the Safety and Efficacy on Progression-Free Survival of RO5072759 + Chlorambucil (GClb) Compared to Rituximab + Chlorambucil (RClb) or Chlorambucil (Clb) Alone in Previously Untreated CLL Patients with Comorbidities (iv) Multicenter Phase I/Ib Study Evaluating the Efficacy and Safety of TGR-1202, a Novel PI3K Delta Inhibitor, in Combination with Obinutuzumab and Chlorambucil in Patients with CLL (v) A Multicenter Phase I/Ib Study Evaluating the Efficacy and Safety of TGR-1202, a Novel PI3K Delta Inhibitor, in Combination with Obinutuzumab and Chlorambucil in Patients with CLL (vi) A Phase 3, Randomized, Open-Label Study Evaluating the Efficacy and Safety of Idelalisib in Combination with Obinutuzumab Compared to Chlorambucil in Combination with Obinutuzumab for Previously Untreated CLL (vii) A Phase 1b Open-Label Study to Evaluate the Safety and Efficacy of TRU-016 in Combination with Rituximab or in Combination with Obinutuzumab in Patients with Chronic Lymphocytic Leukemia (viii) A Phase Ib Study of the Safety and Pharmacology of MPDL3280A Administered with Obinutuzumab in Patients with Relapsed/Refractory Follicular Lymphoma and Diffuse Large B-Cell Lymphoma (ix) A Phase I/II Study of Lenalidomide and Obinutuzumab (GA101) in Relapsed Indolent NHL (x) A Multicenter, Open-Label, Single-Arm, Multiple Dose Study to Assess the Pharmacokinetics of RO5072759 in Chinese Patients with CD20+ Malignant Disease (xi) An Open-Label, Multicentre, Randomized, Phase Ib Study to Investigate the Safety and Efficacy of RO5072759 Given in Combination with CHOP, FC or Bendamustine Chemotherapy in Patients with CD20+ B-Cell Follicular NHL (xii) An Open-Label, Multicenter, Randomized Phase II Trial Comparing the Efficacy, Safety, and Pharmacokinetics of GA101 1000 mg versus 2000 mg in Patients with Previously Untreated CLL (xiii) A Multicenter, Open-Label, Single-Arm, Phase IIIb International Study Evaluating the Safety of Obinutuzumab Alone or in Combination with Chemotherapy in Patients with Previously Untreated or Relapsed/Refractory CLL (xiv) Phase II Trial to Evaluate The Efficacy of Obinutuzumab (RO5072759) + Bendamustine Treatment in Patients with Refractory Or Relapsed CLL (xv) A Phase II, Open-Label, Multicenter Study of Efficacy, Safety, and Biomarkers in Patients with Previously Untreated Advanced DLBCL Treated with GA101 (RO5072759) in Combination with CHOP Chemotherapy (xvi) A Phase IB/II Study of GA101 in Combination with High-Dose Methylprednisolone (HDMP) in CLL Patients (xvii) A Phase Ib Multicenter Dose-Finding And Safety Study Of GDC-0199 and Obinutuzumab in Patients with Relapsed or Refractory or Previously Untreated CLL (xviii) A Phase Ib/II Study of Obinutuzumab Combined to Lenalidomide for the Treatment of Relapsed/Refractory Follicular and Aggressive DLBCL and MCL B-Cell Lymphoma (xix) A Phase III, Multicenter, Open-Label Randomized Trial Comparing the Efficacy of GA101 (RO5072759) in Combination with CHOP (G-CHOP) versus Rituximab and CHOP (R-CHOP) in Previously Untreated Patients with CD20-Positive DLBCL (xx) Phase II Randomized Trial Comparing GA101 (Obinutuzumab) and Rituximab in Patients with Previously Untreated Low Tumor Burden Indolent NHL (xxi) A Multicentre, Phase III, Open-Label, Randomized Study in Previously Untreated Patients with Advanced Indolent NHL Evaluating the Benefit of GA101 (RO5072759) + Chemotherapy Compared to Rituximab + Chemotherapy followed by GA101 or Rituximab Maintenance Therapy in Responders (xxii) An Open-Label, Multicenter, Phase Ib Trial of GA101 (RO5072759) in Combination with Chemotherapy in Patients with Previously Untreated CLL (xxiii) A Phase Ib, Open-Label Study Evaluating the Safety and Pharmacokinetics of GDC-0199 (ABT-199) in Combination with Rituximab (R) or Obinutuzumab (G) Plus Cyclophosphamide, Doxorubicin, Vincristine and Prednisone (Chop) in Patients with B-Cell NHL and DLBCL (xxiv) An Open-Label, Multicenter, Randomized, Phase III Study to Investigate the Efficacy and Safety of Bendamustine Compared with Bendamustine+RO5072759 (GA101) in Patients with Rituximab-Refractory, Indolent NHL
Dacetuzumab	CD40	Humanized	NHL	No information

Lintuzumab	CD33	Humanized	AML, MDS, CML, APL	(i) A Phase I/II Study of Low Dose Cytarabine and Lintuzumab-Ac225 in Older Patients with Untreated AML (ii) Phase I Trial of Targeted Atomic Nano-Generators (Actinium-225-Labeled Humanized Anti-CD33 Monoclonal Antibody HuM195) in Patients with Advanced Myeloid Malignancies

NHL: non-Hodgkin's lymphoma; CLL: chronic lymphocytic leukemia; ALL: acute lymphocytic leukemia; AML: acute myeloid leukemia; ITP: idiopathic thrombocytopenic purpura; DLBCL: diffuse large B-cell lymphoma; MM: multiple myeloma; HL: Hodgkin's lymphoma; APL: acute promyelocytic leukemia.

## References

[1] Walawski J. (2008). Nowe leki i nowe podejście do leczenia a przeżycie chorych na nowotwory układu chłonnego. *Onkologia w Praktyce Klinicznej*.

[2] Wróbel T. (2010). Przeciwciała monoklonalne anty-CD20 w terapii chłoniaków agresywnych. *Hematologia*.

[3] Huszno J., Nowara E., Suwiński R. (2011). Rola polimorfizmów genowych w terapii przeciwnowotworowej ukierunkowanej na cele molekularne—przeciwciała monoklonalne. *Onkologia Polska*.

[4] Powroźnik B., Kubowicz P., Pękala E. (2012). Przeciwciała monoklonalne w terapii celowanej. *Postępy Higieny i Medycyny Doświadczalnej*.

[5] Kobayashi Y. (2011). Molecular target therapy in hematological malignancy: front-runners and prototypes of small molecule and antibody therapy. *Japanese Journal of Clinical Oncology*.

[6] Cang S., Mukhi N., Wang K., Liu D. (2012). Novel CD20 monoclonal antibodies for lymphoma therapy. *Journal of Hematology and Oncology*.

[7] Kirkwood J. M., Butterfield L. H., Tarhini A. A., Zarour H., Kalinski P., Ferrone S. (2012). Immunotherapy of cancer in 2012. *CA: Cancer Journal for Clinicians*.

[8] Tomblyn M. (2012). Radioimmunotherapy for B-cell non-Hodgkin lymphomas. *Cancer Control*.

[9] Chamarthy M. R., Williams S. C., Moadel R. M. (2011). Radioimmunotherapy of non-Hodgkin's lymphoma: from the “magic bullets” to ‘radioactive magic bullets’. *Yale Journal of Biology and Medicine*.

[10] Coiffier B., Lepretre S., Pedersen L. M. (2008). Safety and efficacy of ofatumumab, a fully human monoclonal anti-CD20 antibody, in patients with relapsed or refractory B-cell chronic lymphocytic leukemia: a phase 1-2 study. *Blood*.

[11] Wierda W. G., Padmanabhan S., Chan G. W., Gupta I. V., Lisby S., Österborg A. (2011). Ofatumumab is active in patients with fludarabine-refractory CLL irrespective of prior rituximab: results from the phase 2 international study. *Blood*.

[12] Flinn I. W., Harwin W. N., Ward P. (2012). Phase II Trial of Ofatumumab (OFA) for Older Patients and Patients Who Refuse Fludarabine-Based Regimens with Previously Untreated ChronicLymphocytic Leukemia (CLL) or Small Lymphocytic Lymphoma (SLL). *Blood*.

[13] Badoux X. C., Keating M. J., Wen S. (2013). Phase II study of lenalidomide and rituximab as salvage therapy for patients with relapsed or refractory chronic lymphocytic leukemia. *Journal of Clinical Oncology*.

[14] Wendtner C.-M., Hillmen P., Mahadevan D. (2012). Final results of a multicenter phase 1 study of lenalidomide in patients with relapsed or refractory chronic lymphocytic leukemia. *Leukemia and Lymphoma*.

[15] Czuczman M. S., Fayad L., Delwail V. (2012). Ofatumumab monotherapy in rituximab-refractory follicular lymphoma: results from a multicenter study. *Blood*.

[16] Merli M., Ferrario A., Basilico C. (2013). Novel agents in indolent lymphomas. *Therapeutic Advances in Hematology*.

[17] Srinivasan A., Mukherji S. K. (2011). Tositumomab and iodine I 131 tositumomab (Bexaar). *American Journal of Neuroradiology*.

[18] Dillman R. O. (2006). Radioimmunotherapy of B-cell lymphoma with radiolabelled anti-CD20 monoclonal antibodies. *Clinical and Experimental Medicine*.

[19] Wahl R. L. (2005). Tositumomab and 131I therapy in non-Hodgkin’s lymphoma. *Journal of Nuclear Medicine*.

[20] O'Reilly M. K., Paulson J. C. (2009). Siglecs as targets for therapy in immune-cell-mediated disease. *Trends in Pharmacological Sciences*.

[21] Hoelzer D. (2011). Novel antibody-based therapies for acute lymphoblastic leukemia. *Hematology*.

[22] Ricart A. D. (2011). Antibody-drug conjugates of calicheamicin derivative: gemtuzumab ozogamicin and inotuzumab ozogamicin. *Clinical Cancer Research*.

[23] Zhu X., Ma Y., Liu D. (2010). Novel agents and regimens for acute myeloid leukemia: 2009 ASH annual meeting highlights. *Journal of Hematology and Oncology*.

[24] Duong H. K., Sekeres M. A. (2009). Targeted treatment of acute myeloid leukemia in older adults: role of gemtuzumab ozogamicin. *Clinical Interventions in Aging*.

[25] Nabhan C., Rundhaugen L., Jatoi M. (2004). Gemtuzumab ozogamicin (Mylotarg) is infrequently associated with sinusoidal obstructive syndrome/veno-occlusive disease. *Annals of Oncology*.

[26] Katz J., Janik J. E., Younes A. (2011). Brentuximab vedotin (SGN-35). *Clinical Cancer Research*.

[27] Reichert J. M. (2011). Antibody-based therapeutics to watch in 2011. *mAbs*.

[28] Younes A., Bartlett N. L., Leonard J. P. (2010). Brentuximab vedotin (SGN-35) for relapsed CD30-positive lymphomas. *The New England Journal of Medicine*.

[29] Skotnicki A. B., Nowak W. S., Piotrowska M. (2003). Campath-1H (alemtuzumab)—nowy lek w terapii przewleklej bialaczki limfocytowej. *Acta Hematologica Polonica*.

[30] Lundin J., Kimby E., Björkholm M. (2002). Phase II trial of subcutaneous anti-CD52 monoclonal antibody alemtuzumab (Campath-1H) as first-line treatment for patients with B-cell chronic lymphocytic leukemia (B-CLL). *Blood*.

[31] Hoelzer D. (2011). Novel antibody-based therapies for acute lymphoblastic leukemia. *Hematology/the Education Program of the American Society of Hematology. American Society of Hematology*.

[32] Kirkwood J. M., Butterfield L. H., Tarhini A. A., Zarour H., Kalinski P., Ferrone S. (2012). Immunotherapy of cancer in 2012. *CA Cancer Journal for Clinicians*.

[33] Bello C., Sotomayor E. M. (2007). Monoclonal antibodies for B-cell lymphomas: rituximab and beyond. *Hematology*.

[34] Shi J. D., Bullock C., Hall W. C. (2002). In vivo pharmacodynamic effects of Hu1D10 (Remitogen), a humanized antibody reactive against a polymorphic determinant of HLA-DR expressed on B cells. *Leukemia and Lymphoma*.

[35] Mone A. P., Huang P., Pelicano H. (2004). Hu1D10 induces apoptosis concurrent with activation of the AKT survival pathway in human chronic lymphocytic leukemia cells. *Blood*.

[36] Robak T. (2008). Novel monoclonal antibodies for the treatment of chronic lymphocytic leukemia. *Current Cancer Drug Targets*.

[37] Leonard J. P., Link B. K. (2002). Immunotherapy of non-hodgkin’s lymphoma with hll2 (epratuzumab, an anti-CD22 monoclonal antibody) and Hu I D 1 0 (apolizumab). *Seminars in Oncology*.

[38] Rech J., Repp R., Rech D. (2006). A humanized HLA-DR antibody (hu1D10, apolizumab) in combination with granulocyte colony-stimulating factor (filgrastim) for the treatment of non-Hodgkin's lymphoma: a pilot study. *Leukemia and Lymphoma*.

[39] Dunleavy K. J. (2005). Phase I study of combination of rituximab with apolizumab in relapsed/refractory B-cell lymphoma and chronic lymphocytic leukemia. *Journal of Clinical Oncology*.

[40] Stein R., Mattes M. J., Cardillo T. M. (2007). CD74: a new candidate target for the immunotherapy of B-cell neoplasms. *Clinical Cancer Research*.

[41] Frölich D., Blassfeld D., Reiter K. (2012). The anti-CD74 humanized monoclonal antibody, milatuzumab, which targets the invariant chain of MHC II complexes, alters B-cell proliferation, migration, and adhesion molecule expression. *Arthritis Research &Therapy*.

[42] Starlets D., Gore Y., Binsky I. (2006). Cell-surface CD74 initiates a signaling cascade leading to cell proliferation and survival. *Blood*.

[43] http://www.immunomedics.com/milatuzumab.shtml.

[44] Burton J. D., Ely S., Reddy P. K. (2004). CD74 is expressed by multiple myeloma and is a promising target for therapy. *Clinical Cancer Research*.

[45] Mark T., Martin P., Leonard J. P., Niesvizky R. (2009). Milatuzumab: a promising new agent for the treatment of lymphoid malignancies. *Expert Opinion on Investigational Drugs*.

[46] Binsky I., Haran M., Starlets D. (2007). IL-8 secreted in a macrophage migration-inhibitory factor- and CD74-dependent manner regulates B cell chronic lymphocytic leukemia survival. *Proceedings of the National Academy of Sciences of the United States of America*.

[47] Ong G. L., Goldenberg D. M., Hansen H. J., Mattes M. J. (1999). Cell surface expression and metabolism of major histocompatibility complex class II invariant chain (CD74) by diverse cell lines. *Immunology*.

[48] Sapra P., Stein R., Pickett J. (2005). Anti-CD74 antibody-doxorubicin conjugate, IMMU-110, in a human multiple myeloma xenograft and in monkeys. *Clinical Cancer Research*.

[49] Alinari L., Yu B., Christian B. A. (2011). Combination anti-CD74 (milatuzumab) and anti-CD20 (rituximab) monoclonal antibody therapy has in vitro and in vivo activity in mantle cell lymphoma. *Blood*.

[50] Stein R., Smith M. R., Chen S., Zalath M., Goldenberg D. M. (2009). Combining milatuzumab with bortezomib, doxorubicin, or dexamethasone improves responses in multiple myeloma cell lines. *Clinical Cancer Research*.

[51] Christian B., Alinari L., Earl C. A phase I study of Milatuzumab a humanized anti-CD74 antibody and Veltuzumab, a humanized anti-CD20 antibody, in patients with relapsed and refractory B-Cell Non-Hodgkin's Lymphoma.

[52] Berkova Z., Tao R.-H., Samaniego F. (2010). Milatuzumab-a promising new immunotherapeutic agent. *Expert Opinion on Investigational Drugs*.

[53] Wong B. Y., Dang N. H. (2010). Inotuzumab ozogamicin as novel therapy in lymphomas. *Expert Opinion on Biological Therapy*.

[54] Bello C., Sotomayor E. M. (2007). Monoclonal antibodies for B-cell lymphomas: rituximab and beyond. *Hematology/the Education Program of the American Society of Hematology. American Society of Hematology*.

[55] DiJoseph J. F., Armellino D. C., Boghaert E. R. (2004). Antibody-targeted chemotherapy with CMC-544: a CD22-targeted immunoconjugate of calicheamicin for the treatment of B-lymphoid malignancies. *Blood*.

[56] Thomas D. A. (2012). Inotuzumab: the most active single agent in acute lymphoblastic leukemia?. *Clinical Advances in Hematology and Oncology*.

[57] Advani A., Gisselbrecht E. G., Rohatiner A. (2005). Preliminary report of a phase 1 study of CMC-544, an antibody-targeted chemotherapy agent, in patients with B-cell non Hodgkin’s lymphoma (NHL). *Blood*.

[58] DiJoseph J. F., Dougher M. M., Kalyandrug L. B. (2006). Antitumor efficacy of a combination of CMC-544 (inotuzumab ozogamicin), a CD22-targeted cytotoxic immunoconjugate of calicheamicin, and rituximab against non-Hodgkin's B-cell lymphoma. *Clinical Cancer Research*.

[59] de Vries J. F., Zwaan C. M., de Bie M. (2012). The novel calicheamicin-conjugated CD22 antibody inotuzumab ozogamicin (CMC-544) effectively kills primary pediatric acute lymphoblastic leukemia cells. *Leukemia*.

[60] Kebriaei P., Wilhelm K., Ravandi F. (2013). Feasibility of Allografting in patients with advanced acute lymphoblastic leukemia after salvage therapy with inotuzumab ozogamicin. *Clinical Lymphoma, Myeloma and Leukemia*.

[61] Thomas X. (2012). Inotuzumab ozogamicin in the treatment of B-cell acute lymphoblastic leukemia. *Expert Opinion on Investigational Drugs*.

[62] Fayad L., Offner F., Smith M. R. (2013). Safety and clinical activity of a combination therapy comprising two antibody-based targeting agents for the treatment of non-hodgkin lymphoma: results of a phase I/II study evaluating the immunoconjugate inotuzumab ozogamicin with rituximab. *Journal of Clinical Oncology*.

[63] Ogura M., Hatake K., Ando K. (2012). Phase I study of anti-CD22 immunoconjugate inotuzumab ozogamicin plus rituximab in relapsed/refractory B-cell non-Hodgkin lymphoma. *Cancer Science*.

[64] Furman R. R., Coleman M., Leonard J. P. (2004). Epratuzumab in non-Hodgkin's lymphomas. *Current Treatment Options in Oncology*.

[65] Leonard J. P., Goldenberg D. M. (2007). Preclinical and clinical evaluation of epratuzumab (anti-CD22 IgG) in B-cell malignancies. *Oncogene*.

[66] Strauss S. J., Morschhauser F., Rech J. (2006). Multicenter phase II trial of immunotherapy with the humanized anti-CD22 antibody, epratuzumab, in combination with rituximab, in refractory or recurrent non-Hodgkin's lymphoma. *Journal of Clinical Oncology*.

[67] Leonard J. P., Schuster S. J., Emmanouilides C. (2008). Durable complete responses from therapy with combined epratuzumab and rituximab: final results from an international multicenter, phase 2 study in recurrent, indolent, non-Hodgkin lymphoma. *Cancer*.

[68] Rossi E. A., Goldenberg D. M., Cardillo T. M., Stein R., Chang C.-H. (2009). Hexavalent bispecific antibodies represent a new class of anticancer therapeutics: 1. Properties of anti-CD20/CD22 antibodies in lymphoma. *Blood*.

[69] Micallef I. N. M., Maurer M. J., Wiseman G. A. (2011). Epratuzumab with rituximab, cyclophosphamide, doxorubicin, vincristine, and prednisone chemotherapy in patients with previously untreated diffuse large B-cell lymphoma. *Blood*.

[70] Morschhauser F., Kraeber-Bodéré F., Wegener W. A. (2010). High rates of durable responses with anti-CD22 fractionated radioimmunotherapy: results of a multicenter, phase I/II study in non-Hodgkin's lymphoma. *Journal of Clinical Oncology*.

[71] Kasner M. T. (2010). Novel targets for treatment of adult acute lymphocytic leukemia. *Current Hematologic Malignancy Reports*.

[72] Raetz E. A., Cairo M. S., Borowitz M. J. (2008). Chemoimmunotherapy reinduction with epratuzumab in children with acute lymphoblastic leukemia in marrow relapse: a children's oncology group pilot study. *Journal of Clinical Oncology*.

[73] Dörner T., Shock A., Smith K. G. C. (2012). CD22 and autoimmune disease. *International Reviews of Immunology*.

[74] Leonard J., Coleman M. P., Ketas J. (2004). Epratuzumab, a humanized anti-CD22 antibody, in aggressive non-Hodgkin's lymphoma: phase I/II clinical trial results. *Clinical Cancer Research*.

[75] Dörner T., Goldenberg D. M. (2007). Targeting CD22 as a strategy for treating systemic autoimmune diseases. *Therapeutics and Clinical Risk Management*.

[76] Leonard J. P., Coleman M., Ketas J. (2005). Combination antibody therapy with epratuzumab and rituximab in relapsed or refractory non-Hodgkin's lymphoma. *Journal of Clinical Oncology*.

[77] Reichert J. M. (2011). Antibody-based therapeutics to watch in 2011. *MAbs*.

[78] Niederfellner G., Lammens A., Mundigl O. (2011). Epitope characterization and crystal structure of GA101 provide insights into the molecular basis for type I/II distinction of CD20 antibodies. *Blood*.

[79] Pauwels P. J., Dumontet C., Reichert J. M. (2012). 7th cancer scientific forum of the Cancéropôle Lyon Auvergne Rhône-Alpes. *mAbs*.

[80] Goede V., Fischer K., Busch R. (2014). Obinutuzumab plus chlorambucil in patients with CLL and coexisting conditions. *The New England Journal of Medicine*.

[81] Goldenberg D. M., Rossi E. A., Stein R. (2009). Properties and structure-function relationships of veltuzumab (hA20), a humanized anti-CD20 monoclonal antibody. *Blood*.

[82] Negrea G. O., Elstrom R., Allen S. L. (2011). Subcutaneous injections of low-dose veltuzumab (humanized anti-CD20 antibody) are safe and active in patients with indolent non-Hodgkin's lymphoma. *Haematologica*.

[83] Rezvani A. R., Maloney D. G. (2011). Rituximab resistance. *Best Practice and Research: Clinical Haematology*.

[84] Morschhauser F., Marlton P., Vitolo U. (2010). Results of a phase I/II study of ocrelizumab, a fully humanized anti-CD20 mAb, in patients with relapsed/refractory follicular lymphoma. *Annals of Oncology*.

[85] Moore G. L., Chen H., Karki S., Lazar G. A. (2010). Engineered Fc variant antibodies with enhanced ability to recruit complement and mediate effector functions. *mAbs*.

[86] Fournier S., Delespesse G., Rubio M., Biron G., Sarfati M. (1992). CD23 antigen regulation and signaling in chronic lymphocytic leukemia. *Journal of Clinical Investigation*.

[87] Pathan N. I., Chu P., Hariharan K., Cheney C., Molina A., Byrd J. (2008). Mediation of apoptosis by and antitumor activity of lumiliximab in chronic lymphocytic leukemia cells and CD23^+^ lymphoma cell lines. *Blood*.

[88] Byrd J. C., O'Brien S., Flinn I. W. (2007). Phase 1 study of lumiliximab with detailed pharmacokinetic and pharmacodynamic measurements in patients with relapsed or refractory chronic lymphocytic leukemia. *Clinical Cancer Research*.

[89] Byrd J. C., Kipps T. J., Flinn I. W. (2010). Phase 1/2 study of lumiliximab combined with fludarabine, cyclophosphamide, and rituximab in patients with relapsed or refractory chronic lymphocytic leukemia. *Blood*.

[90] Tourangeau L. M., Kavanaugh A., Wasserman S. I. (2011). The role of monoclonal antibodies in the treatment of severe asthma. *Therapeutic Advances in Respiratory Disease*.

[91] Poole J. A., Meng J., Reff M., Spellman M. C., Rosenwasser L. J. (2005). Anti-CD23 monoclonal antibody, lumiliximab, inhibited allergen-induced responses in antigen-presenting cells and T cells from atopic subjects. *The Journal of Allergy and Clinical Immunology*.

[92] Veliz M., Pinilla-Ibarz J. (2012). Treatment of relapsed or refractory chronic lymphocytic leukemia. *Cancer Control*.

[93] Chambers C. A., Allison J. P. (1999). Costimulatory regulation of T cell function. *Current Opinion in Cell Biology*.

[94] Czuczman M. S. (2005). Anti-CD80 monoclonal antibody: galiximab. *Haematologica Reports*.

[95] Plumas J., Chaperot L., Jacob M.-C. (1995). Malignant B lymphocytes from non-Hodgkin's lymphoma induce allogeneic proliferative and cytotoxic T cell responses in primary mixed lymphocyte cultures: an important role of costimulatory molecules CD80 (B7-1) and CD86 (B7-2) in stimulation by tumor cells. *European Journal of Immunology*.

[96] Martinez-Paniagua M. A., Vega M. I., Huerta-Yepez S. (2012). Galiximab signals B-NHL cells and inhibits the activities of NF-*κ*B-induced YY1- and snail-resistant factors: mechanism of sensitization to apoptosis by chemoimmunotherapeutic drugs. *Molecular Cancer Therapeutics*.

[97] Bhat S., Czuczman M. S. (2010). Galiximab: a review. *Expert Opinion on Biological Therapy*.

[98] Smith S. M., Schöder H., Johnson J. L., Jung S.-H., Bartlett N. L., Cheson B. D. (2013). The anti-CD80 primatized monoclonal antibody, galiximab, is well-tolerated but has limited activity in relapsed Hodgkin lymphoma: cancer and Leukemia Group B 50602 (Alliance). *Leukemia and Lymphoma*.

[99] Czuczman M. S., Thall A., Witzig T. E. (2005). Phase I/II study of galiximab, an anti-CD80 antibody, for relapsed or refractory follicular lymphoma. *Journal of Clinical Oncology*.

[100] Czuczman M. S., Leonard J. P., Jung S. (2012). Phase II trial of galiximab (anti-CD80 monoclonal antibody) plus rituximab (CALGB 50402): follicular lymphoma international prognostic index (FLIPI) score is predictive of upfront immunotherapy responsiveness. *Annals of Oncology*.

[101] Gottlieb A. B., Kang S., Linden K. G. (2004). Evaluation of safety and clinical activity of multiple doses of the anti-CD80 monoclonal antibody, galiximab, in patients with moderate to severe plaque psoriasis. *Clinical Immunology*.

[102] Feldman E. J., Kalaycio M., Weiner G. (2003). Treatment of relapsed or refractory acute myeloid leukemia with humanized anti-CD33 monoclonal antibody HuM195. *Leukemia*.

[103] Feldman E. J., Brandwein J., Stone R. (2005). Phase III randomized multicenter study of a humanized anti-CD33 monoclonal antibody, lintuzumab, in combination with chemotherapy, versus chemotherapy alone in patients with refractory or first-relapsed acute myeloid leukemia. *Journal of Clinical Oncology*.

[104] Sutherland M. K., Yu C., Anderson M. (2010). 5-azacytidine enhances the anti-leukemic activity of lintuzumab (SGN-33) in preclinical models of acute myeloid leukemia. *mAbs*.

[105] Miederer M., McDevitt M. R., Sgouros G., Kramer K., Cheung N.-K. V., Scheinberg D. A. (2004). Pharmacokinetics, dosimetry, and toxicity of the targetable atomic generator, 225Ac-HuM195, in nonhuman primates. *Journal of Nuclear Medicine*.

[106] Rosenblat T. L., McDevitt M. R., Mulford D. A. (2010). Sequential cytarabine and *α*-particle immunotherapy with bismuth-213-lintuzumab (HuM195) for acute myeloid leukemia. *Clinical Cancer Research*.

[107] Schwartz M. A., Lovett D. R., Redner A. (1993). Dose-escalation trial of M195 labeled with iodine 131 for cytoreduction and marrow ablation in relapsed or refractory myeloid leukemias. *Journal of Clinical Oncology*.

[108] Borthakur G., Rosenblum M. G., Talpaz M. (2013). Phase 1 study of an anti-CD33 immunotoxin, humanized monoclonal antibody M195 conjugated to recombinant gelonin (HUM-195/rGEL), in patients with advanced myeloid malignancies. *Haematologica*.

[109] Van Kooten G., Banchereau J. (2000). CD40-CD40 ligand. *Journal of Leukocyte Biology*.

[110] Tong A. W., Stone M. J. (2003). Prospects for CD40-directed experimental therapy of human cancer. *Cancer Gene Therapy*.

[111] Khubchandani S., Czuczman M. S., Hernandez-Ilizaliturri F. J. (2009). Dacetuzumab, a humanized mAb against CD40 for the treatment of hematological malignancies. *Current Opinion in Investigational Drugs*.

[112] Advani R., Forero-Torres A., Furman R. R. (2009). Phase I study of the humanized anti-CD40 monoclonal antibody dacetuzumab in refractory or recurrent non-Hodgkin's lymphoma. *Journal of Clinical Oncology*.

[113] Forero-Torres A., Bartlett N., Beaven A. (2013). Pilot study of dacetuzumab in combination with rituximab and gemcitabine for relapsed or refractory diffuse large B-cell lymphoma. *Leukemia and Lymphoma*.

[114] Tai Y.-T., Li X.-F., Catley L. (2005). Immunomodulatory drug lenalidomide (CC-5013, IMiD3) augments anti-CD40 SGN-40-induced cytotoxicity in human multiple myeloma: clinical implications. *Cancer Research*.

[115] Lapalombella R., Gowda A., Joshi T. (2009). The humanized CD40 antibody SGN-40 demonstrates pre-clinical activity that is enhanced by lenalidomide in chronic lymphocytic leukaemia. *British Journal of Haematology*.

[116] Agura E., Niesvizky R., Matous J. (2009). Dacetuzumab (SGN-40), lenalidomide, and weekly dexamethasone in relapsed or refractory multiple myeloma: multiple responses observed in a phase 1b study. *Blood*.

[117] http://clinicaltrials.gov/show/NCT00664898.

[118] Luqman M., Klabunde S., Lin K. (2008). The antileukemia activity of a human anti-CD40 antagonist antibody, HCD122, on human chronic lymphocytic leukemia cells. *Blood*.

[119] Freedman A., Kuruvilla J S., Assouline S. Clinical Activity of Lucatumumab (HCD122) In Patients (pts) with Relapsed/Refractory Hodgkin or Non-Hodgkin Lymphoma Treated In a Phase Ia/II Clinical Trial (NCT00670592). NCT00670592.

[120] Byrd J. C., Kipps T. J., Flinn I. W. (2012). Phase i study of the anti-CD40 humanized monoclonal antibody lucatumumab (HCD122) in relapsed chronic lymphocytic leukemia. *Leukemia and Lymphoma*.

[121] Bensinger W., Maziarz R. T., Jagannath S. (2012). A phase 1 study of lucatumumab, a fully human anti-CD40 antagonist monoclonal antibody administered intravenously to patients with relapsed or refractory multiple myeloma. *British Journal of Haematology*.

[122] http://clinicaltrials.gov/ct2/show/NCT01275209.

[123] http://clinicaltrials.gov/show/NCT00670592.

[124] Fanale M., Assouline S., Kuruvilla J. (2014). Phase IA/II, multicentre, open-label study of the CD40 antagonistic monoclonal antibody lucatumumab in adult patients with advanced non-Hodgkin or Hodgkin lymphoma. *British Journal of Haematology*.

[125] Seiler T. M., Hiddemann W. (2012). Advances in the management of follicular lymphoma. *Current Opinion in Oncology*.

[126] Nagorsen D., Baeuerle P. A. (2011). Immunomodulatory therapy of cancer with T cell-engaging BiTE antibody blinatumomab. *Experimental Cell Research*.

[127] Wang K., Wei G., Liu D. (2012). CD19: a biomarker for B cell development, lymphoma diagnosis and therapy. *Experimental Hematology & Oncology*.

[128] d'Argouges S., Wissing S., Brandl C. (2009). Combination of rituximab with blinatumomab (MT103/MEDI-538), a T cell-engaging CD19-/CD3-bispecific antibody, for highly efficient lysis of human B lymphoma cells. *Leukemia Research*.

[129] Background information for the pediatric subcommittee of the oncologic drugs advisory committee meeting.

[130] Topp M. S., Goekbuget N., Zugmaier G. (2011). Anti-CD19 BiTE blinatumomab induces high complete remission rate in adult patients with relapsed b-precursor all: updated results of an ongoing phase II trial. *ASH Annual Meeting Abstracts*.

[131] Topp M. S., Kufer P., Gökbuget N. (2011). Targeted therapy with the T-cell—engaging antibody blinatumomab of chemotherapy-refractory minimal residual disease in B-lineage acute lymphoblastic leukemia patients results in high response rate and prolonged leukemia-free survival. *Journal of Clinical Oncology*.

[132] Topp M. S., Gökbuget N., Zugmaier G. (2012). Long-term follow-up of hematologic relapse-free survival in a phase 2 study of blinatumomab in patients with MRD in B-lineage ALL. *Blood*.

[133] Grothey A., Galanis E. (2009). Targeting angiogenesis: progress with anti-VEGF treatment with large molecules. *Nature Reviews Clinical Oncology*.

[134] http://www.cancer.gov/cancertopics/druginfo/fda-bevacizumab.

[135] Odia Y., Shih J. H., Kreisl T. N., Fine H. A. (2014). Bevacizumab-related toxicities in the National Cancer Institute malignant glioma trial cohort. *Journal of Neuro-Oncology*.

[136] Ossenkoppele G. J., Stussi G., Maertens J. (2012). Addition of bevacizumab to chemotherapy in acute myeloid leukemia at older age: a randomized phase 2 trial of the Dutch-Belgian Cooperative Trial Group for Hemato-Oncology (HOVON) and the Swiss Group for Clinical Cancer Research (SAKK). *Blood*.

[137] Karp J. E., Gojo I., Pili R. (2004). Targeting vascular endothelial growth factor for relapsed and refractory adult acute myelogenous leukemias: Therapy with sequential 1-*β*-D-arabinofuranosylcytosine, mitoxantrone, and bevacizumab. *Clinical Cancer Research*.

[138] Reiners K. S., Gossmann A., Von Strandmann E. P., Böll B., Engert A., Borchmann P. (2009). Effects of the anti-VEGF Monoclonal antibody bevacizumab in a preclinical model and in patients with refractory and multiple relapsed hodgkin lymphoma. *Journal of Immunotherapy*.

[139] Foss H., Araujo I. D., Demel G. (1997). Expression of vascular endothelial growth factor in lymphomas and Castleman’s disease. *The Journal of Pathology*.

[140] Bertolini F., Paolucci M., Peccatori F. (1999). Angiogenic growth factors and endostatin in non-Hodgkin’s lymphoma. *British Journal of Haematology*.

[141] Salven P., Teerenhovi L., Joensuu H. (1997). A high pretreatment serum vascular endothelial growth factor concentration is associated with poor outcome in non-Hodgkin's lymphoma. *Blood*.

[142] Avramis I. A., Panosyan E. H., Dorey F., Holcenberg J. S., Avramis V. I. (2006). Correlation between high vascular endothelial growth factor-A serum levels and treatment outcome in patients with standard-risk acute lymphoblastic leukemia: a report from Children’s Oncology Group Study CCG-1962. *Clinical Cancer Research*.

[143] Ganjoo K., An C., Robertson M. (2006). Rituximab, Bevacizumab and CHOP (RA-CHOP) in untreated diffuse large B-cell lymphoma: Safety, biomarker and pharmacokinetic analysis. *Leukemia and Lymphoma*.

[144] Stopeck A. T., Unger J. M., Rimsza L. M. (2012). A phase 2 trial of standard-dose cyclophosphamide, doxorubicin, vincristine, prednisone (CHOP) and rituximab plus bevacizumab for patients with newly diagnosed diffuse large B-cell non-Hodgkin lymphoma: SWOG 0515. *Blood*.

[145] http://clinicaltrials.gov/ct2/show/NCT00096148.

[146] Wang L., Shi W.-Y., Yang F. (2011). Bevacizumab potentiates chemotherapeutic effect on t-leukemia/lymphoma cells by direct action on tumor endothelial cells. *Haematologica*.

[147] Bujanda D. A. (2008). Complete response of relapsed angioimmunoblastic T-cell lymphoma following therapy with bevacizumab. *Annals of Oncology*.

